# Prognostication of end-stage heart failure using a machine learning model based on haemodynamics and cardiac energetics data

**DOI:** 10.1093/ehjdh/ztag162

**Published:** 2026-11-13

**Authors:** Pablo J Blanco, Carlos A Bulant, Rohan Goswami, Ryo Torii, Evan L Brittain, Jeffrey S Annis, Mark N Belkin, Héctor M García-García, Christos Bourantas, Jonathan Grinstein

**Affiliations:** Department of Mathematical and Computational Methods, National Laboratory for Scientific Computing, Petrópolis 25651-075, Brazil; Instituto Pladema, Universidad Nacional del Centro de la Provincia de Buenos Aires (UNICEN), Tandil, Bs.As., 7000, Argentina; Consejo Nacional de Investigaciones Científicas y Técnicas (CONICET), Tandil, Bs.As., 7000, Argentina; Division of Advanced Heart Failure and Transplant Cardiology, Mayo Clinic, Jacksonville, FL 32246, USA; Department of Mechanical Engineering, University College London, London WC1E 7JE, UK; Division of Cardiovascular Medicine, Vanderbilt University Medical Center, Nashville, TN 37232, USA; Vanderbilt Institute for Clinical and Translational Research, Vanderbilt University Medical Center, Nashville, TN 37203, USA; Division of Cardiovascular Medicine, Vanderbilt University Medical Center, Nashville, TN 37232, USA; Vanderbilt Institute for Clinical and Translational Research, Vanderbilt University Medical Center, Nashville, TN 37203, USA; Department of Medicine, Section of Cardiology, University of Chicago, Chicago, IL 60637, USA; Medstar Washington Hospital Center, Section of Interventional Cardiology, Washington, DC 20010, USA; Department of Cardiology, Barts Heart Center, London EC1A 7BE, UK; Department of Medicine, Section of Cardiology, University of Chicago, Chicago, IL 60637, USA

**Keywords:** Energetics, Heart failure, Haemodynamics, Machine learning

## Abstract

**Aims:**

In the evolving landscape of heart failure treatment, clinicians face challenges in predicting which end-stage heart failure patients will require heart replacement therapies, such as left ventricular assist devices or transplantation, or are at risk of death. Traditional risk models often fail to accurately stratify patients due to complex interactions of guideline-directed medical therapy, individual responses, and haemodynamic or shock phenotypes.

**Methods and results:**

This study introduces a novel approach using machine learning to predict patient outcomes by analysing a comprehensive set of haemodynamic variables, including metrics tied to cardiac energetics—an emerging field focused on heart energy dynamics during failure. A dataset of 2904 patients across the spectrum of heart failure was used to train and test the models, which underwent rigorous model selection, cross-validation, and hyperparameter tuning on 70% of the data, using forward feature selection. Metrics for the remaining 30% used for testing resulted in an area under the curve of 0.81, a Matthews correlation coefficient of 0.35, and an accuracy of 0.74 to predict the composite endpoint of survival free from death, left ventricular assist devices, or transplant at 1 year. We also demonstrate consistency across the different patient databases used for training, validation, and testing, supported by an F1 score of 0.65 overall and 0.82 for patients who received medical management. The model shows a promising negative predictive value of 0.92, but external validation with a prospective database is needed before clinical application.

**Conclusion:**

This approach is novel in integrating haemodynamics and cardiac energetics with machine learning towards more individualized and effective treatment for heart failure patients.

## Graphical Abstract

**Graphical Abstract:**
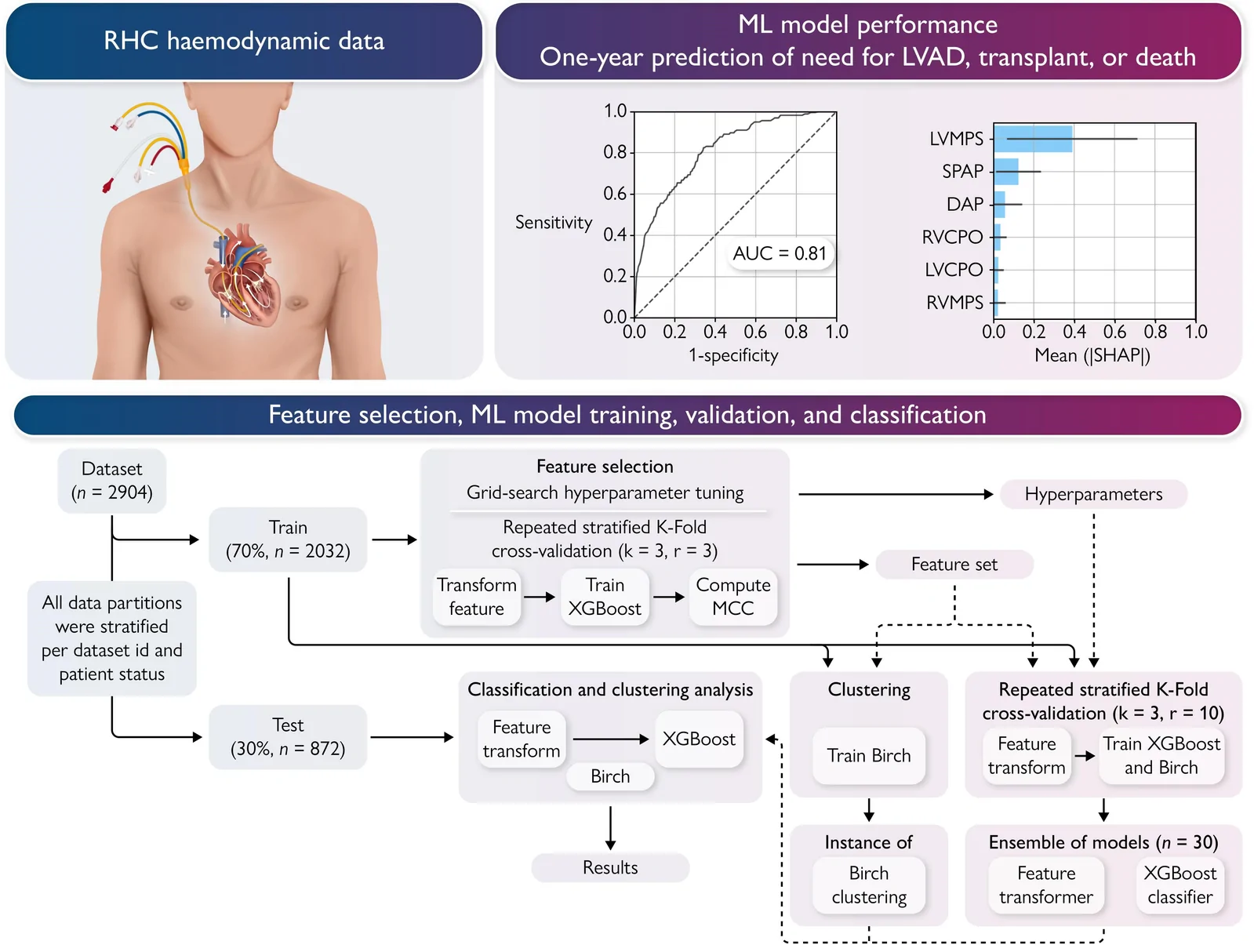

## Introduction

Heart failure is a leading cause of morbidity and mortality worldwide, and its management remains a clinical challenge despite advancements in medical and device therapies. Assessing heart function and determining the best treatment for patients with end-stage heart failure typically involves a range of diagnostic tools, including right heart catheterization (RHC) and measurements of cardiac output, intra-cardiac and pulmonary pressures, and other haemodynamic parameters. However, the complexity of these data points often makes it difficult for clinicians to accurately predict the course of the disease and the need for advanced treatments, such as left ventricular assist devices (LVAD) or heart transplantation (HT). Recently, the field of advanced haemodynamics has emerged as a powerful prognostic tool for heart failure patients. Whereas traditional haemodynamic variables reflect the final output of the heart and fail to fully capture the physiologic drivers of patient-specific pathophysiology and thus represent an incomplete assessment of patient risk, advanced haemodynamic variables reflect the underlying mechanical and electrical properties of the heart and better reflect the dynamic state of the cardiac condition. Using advanced hemodynamics, a patient’s myocardial performance profile and energetic phenotype can be determined, which can help define both a patient’s natural history and anticipated response to therapy.^1^

Applied artificial intelligence (AI) and machine learning (ML) offer new ways to process and interpret large volumes of clinical data, providing previously unattainable insights with traditional models and clinical gestalt. ML, in particular, excels at recognizing patterns within data and making predictions based on those patterns. In the context of heart failure, ML can be used to analyse haemodynamic data to predict patient outcomes, such as the likelihood of requiring more advanced treatments or even the risk of death.^2^

This paper introduces a novel AI-driven approach that applies ML algorithms to cardiac energetics—the assessment of the dynamic energy consumption within the heart. Specifically, we use logistic regression (LR), and also the algorithm called eXtreme Gradient Boosting (XGBoost),^3^ which is a powerful decision-tree-based technique to stratify patients based on a wide range of haemodynamic, clinical, and energetic variables. By integrating haemodynamic and energetic data from several large patient databases, we developed a model that can predict the composite endpoint of HT, LVAD implantation, or death (D) at 1 year.

Haemodynamic data are hard to be correctly interpreted in the clinic, and this has led to delays in timely referral for downstream interventions.^4^ The strength of our algorithm is that it accurately extracts the relevant, prognostic variables from the standard haemodynamic inputs and improves risk stratification compared to standard haemodynamic inputs. To better reflect this, we also assessed the predictive performance of our proposed model compared to standard and advanced haemodynamic inputs using logistic regression models.

After proper external validation in prospective cohorts of heart failure patients, this line of research has the potential to transform how we assess cardiac function, moving beyond the traditional haemodynamic assessment, which fails to fully capture patient risk, to personalized care models that reflect the intricate energy dynamics of the failing heart.

## Materials and methods

### Patient population

We jointly consider three databases: the PRognostic Evaluation During Invasive CaTheterization for Heart Failure (PREDICT-HF) cohort,^5^ the University of Chicago Medical Center shock (UCMC-SHOCK) cohort,^6^ and the Vanderbilt University Medical Center (VUMC) cohort.^7^ All three databases include RHC data at baseline before any device or inotropic therapies were initiated, and robust clinical adjudication of cardiac events at 1-year follow-up.

These datasets were included to capture a wide spectrum of heart failure presentations. The VUMC cohort includes all-comers to the cardiac catheterization lab and reflects a lower acuity patient population. The PREDICT-HF cohort similarly includes all-comers to the catheterization lab but notably reflects patients who were referred to the end-stage heart failure service line with a higher pre-test probability of needing advanced therapies. The UCMC-SHOCK cohort includes patients who presented in SCAI stage C shock at the time of presentation to the cardiac catheterization lab, and all patients met institutional criteria for cardiogenic shock including a pulmonary capillary wedge pressure (PCWP)>15mmHg and/or mean pulmonary artery pressure (PA)>25mmHg and a cardiac index $(\text{CI})<2.2\;{\text{L/min/m}}^{2}$.

Stable patients with no need for escalation of support were classified as medical management (MM). Patients with more advanced disease and progressive heart failure symptoms that require advanced therapies (AT), which could be a left ventricle assist device (LVAD) implantation or a HT. The primary composite end-point included LVAD, HT, or D within 1 year. For this work, we dichotomized the target variable as condition negative if the patient status is MM or condition positive if the patient either received AT or died. In this document, hereafter, we refer to the positive condition class as AT/D.

This study involved 2904 patients. Variable distributions are provided in *Table 1*. Fundamental haemodynamic variables are: mean right atrial pressure (MRAP), diastolic pulmonary artery pressure (DPAP), systolic pulmonary artery pressure (SPAP), PCWP, diastolic arterial pressure (DAP), systolic arterial pressure (SAP), and cardiac output (CO). Other derived haemodynamic variables are mean pulmonary pressure (MPAP) and mean arterial pressure (MAP). In turn, energetic variables are aortic pulsatility index (API), left ventricle cardiac power output score (LVCPO), left ventricle myocardial performance score (LVMPS), pulmonary artery pulsatility index (PAPI), right ventricle cardiac power output score (RVCPO), right ventricle myocardial performance score (RVMPS), systemic vascular resistance (SVR), and pulmonary vascular resistance (PVR). Mathematical formulae for the derived haemodynamic and energetic variables are provided in Appendix 2.

**Table 1:** Demographics and patient characteristics are segregated per database

Variable	PREDICT-HF	UCMC-SHOCK	VUMC	All
	(*n* = 594)	(*n* = 193)	(*n* = 2117)	(*n* = 2904)
SPAP	43 (32,60)	60 (46,67)	49 (35,68)	49 (35,65)
DPAP	20 (15,30)	30 (24,37)	22 (16,29)	23 (16,30)
MRAP	10 (6,15)	13 (9,18)	9 (5,13)	9 (6,14)
SAP	117 (105,135)	106 (98,119)	123 (108,139)	120 (106,137)
DAP	70 (63,78)	71 (64,78)	73 (63,82)	72 (63,81)
PCWP	20 (13,25)	25 (20,30)	15 (10,21)	16 (11,23)
CO	5.04 (4.09,6.16)	3.6 (3.03,4.3)	4.87 (3.83,6.03)	4.8 (3.8,5.97)
SVR	15 (11.9,18.3)	23.13 (19.35,28.8)	16.49 (13.07,21.37)	16.49 (13.06,21.37)
PVR	1.84 (1.22,2.88)	3.48 (2.22,4.39)	2.59 (1.41,5.07)	2.41 (1.4,4.42)
API	2.59 (1.67,4)	1.4 (1.03,2.05)	3.6 (2.25,5.5)	3.17 (1.91,5)
LVCPO	0.96 (0.75,1.24)	0.68 (0.56,0.82)	0.95 (0.74,1.22)	0.93 (0.72,1.2)
LVMPS	1.29 (0.67,2.28)	0.48 (0.31,0.75)	1.76 (0.95,3)	1.52 (0.78,2.73)
PAPI	2.22 (1.55,3.28)	2.21 (1.41,3)	3.38 (2,5.57)	2.89 (1.83,4.88)
RVCPO	0.31 (0.23,0.41)	0.3 (0.24,0.37)	0.33 (0.24,0.44)	0.32 (0.24,0.43)
RVMPS	1.08 (0.7,1.7)	1.01 (0.66,1.59)	1.61 (0.9,3.05)	1.42 (0.81,2.59)
1-year status				
MM	414 (69.7%)	59 (30.6%)	1921 (90.7%)	2394 (82.4%)
AT/D	180 (30.3%)	134 (69.4%)	196 (9.3%)	510 (17.6%)
LVAD	35 (19.4%)	54 (40.3%)	48 (24.5%)	137 (26.9%)
HT	24 (13.3%)	46 (34.3%)	148 (75.5%)	218 (42.7%)
Death	121 (67.2%)	34 (25.4%)	0 (0%)	155 (30.4%)
CO method				
Fick	594 (100%)	193 (100%)	1980 (93.5%)	2767 (95.3%)
Thermo	0 (0%)	0 (0%)	137 (6.5%)	137 (4.7%)

See abbreviations in Appendix 1 and mathematical definitions in Appendix 2. Values are median (Q1, Q3), only DPAP, SPAP, and PCWP for the UCMC-SHOCK dataset passed the normality test (p>0.01).

Patients with recorded CO≥10L/min were excluded. All patients receiving inotrope therapy were excluded. From the VUMC dataset, there are no records for inotrope therapy. A total of 594, 193, and 2117 patients from the PREDICT-HF, UCMC-SHOCK, and VUMC datasets were used in this study. The CO in all patients from the PREDICT-HF and UCMC-SHOCK datasets was measured using the Fick method. For the VUMC, we picked CO values acquired by the Fick method whenever available. Otherwise, thermodilution CO data were used. Notably, in the VUMC dataset all patients were alive at 1-year follow-up.

We note that the PREDICT-HF database displays a balanced mix of HFrEF and HFpEF patients, while the UCMC-SHOCK is mostly composed by HFrEF patients. Data for EF in the VUMC database were not available.

### Machine learning model

Assessment and selection of an appropriate machine learning model were critical in handling the high dimensionality and complex interactions of our datasets, as well as providing transparency to the end-user (e.g. clinician) for the management of patients with heart failure. We evaluated multiple models, including Random Forest, support vector machine, multi-layer perceptron, and XGBoost.

Specifically, we exhaustively assessed the predictive performance of logistic regression models when using standard and advanced haemodynamic inputs solely, and compared with the XGBoost model. After comparing model performances (see Appendix 3), we selected the XGBoost approach due to its strong predictive accuracy and robustness to overfitting. XGBoost is an ensemble learning method based on gradient boosting that constructs a sequence of decision trees to improve predictive performance. Each decision tree is trained to model the residual errors present in the current ensemble by minimizing a differentiable loss function. This iterative process aims at progressively reducing prediction error, while regularization mechanisms help control model complexity and prevent overfitting. The final model combines many weak learners into a single, highly accurate predictor.

Ultimately, our final model choice—an ensemble of 30 XGBoost classifiers–was optimized through grid search, with hyperparameters fine-tuned to balance predictive performance and generalizability. This approach allowed us to leverage ensemble averaging to improve stability and confidence in predictions while maintaining interpretability through SHapley Additive exPlanations (SHAP)-based feature analysis. SHAP is a model-agnostic framework aimed at improving the interpretability of individual predictions by quantifying each input feature’s contribution. Grounded in cooperative game theory, it assigns each feature a so-called Shapley value that represents its average marginal contribution across all possible feature combinations. These values indicate how much each feature shifts a prediction relative to a baseline (typically the average prediction), enabling consistent and locally accurate explanations.

We use an ensemble of 30 XGBoost classification models, each trained with different subsamples of the training set, see Section ‘Sample partitioning and training methodology’. Ensemble operation consists of averaging the prediction score (class probability) among models for the population of predictions. Hyperparameters were defined through a grid search during training. The final XGBoost setup comprised 32 estimators with a maximum depth of four levels. We used oversampling with a ratio of 20 for the minority class (1-year status AT/D). The complete list of hyperparameters and the grid-search ranges are detailed in Appendix 5. Each model outputs the class membership probability, which is then ensembled (averaged) and thresholded (value of 0.5) to establish the class. Changing the threshold value allows us to compute the ROC curve and the area under the curve (AUC) metric.

Energetic phenotyping and patient risk stratification can be performed using single energetic variables, such as API, a marker of cardiac efficiency and ventricular-aortic coupling, and LVCPO, a marker of cardiac power. In this two-dimensional space, four quadrants can be defined by the combination of low and high values of API and CPO, reflecting the power-efficiency balance of the patient.^4^ We propose an alternative approach by exploiting an unsupervised automatic clustering in a higher-dimensional space with all the features picked by the forward feature selection procedure (see next section). So, using the complete training set, we trained a balanced iterative reducing and clustering using hierarchies model (BIRCH, see Ref.^8^) to find four clusters (see also Appendix 4). The four clusters are selected to reflect the four quadrants of the API-CPO space, categorized by low–high values. The complete computational methodology is illustrated in *Figure 1*, and details about the training methodology are provided in Section ‘Sample partitioning and training methodology’. It is noteworthy that the feature transformers (power transform) are trained for each XGBoost model on the corresponding training data (fold/repetition) and then used directly to scale the test data. As well, oversampling is only used during training. On the other hand, the feature set and model hyperparameters are shared over all 30 XGBoost models, which differ in the training data, i.e. folds/repetitions over the repeated stratified cross-validation process. This way, we ensure a robust training pipeline.

**Figure 1:**
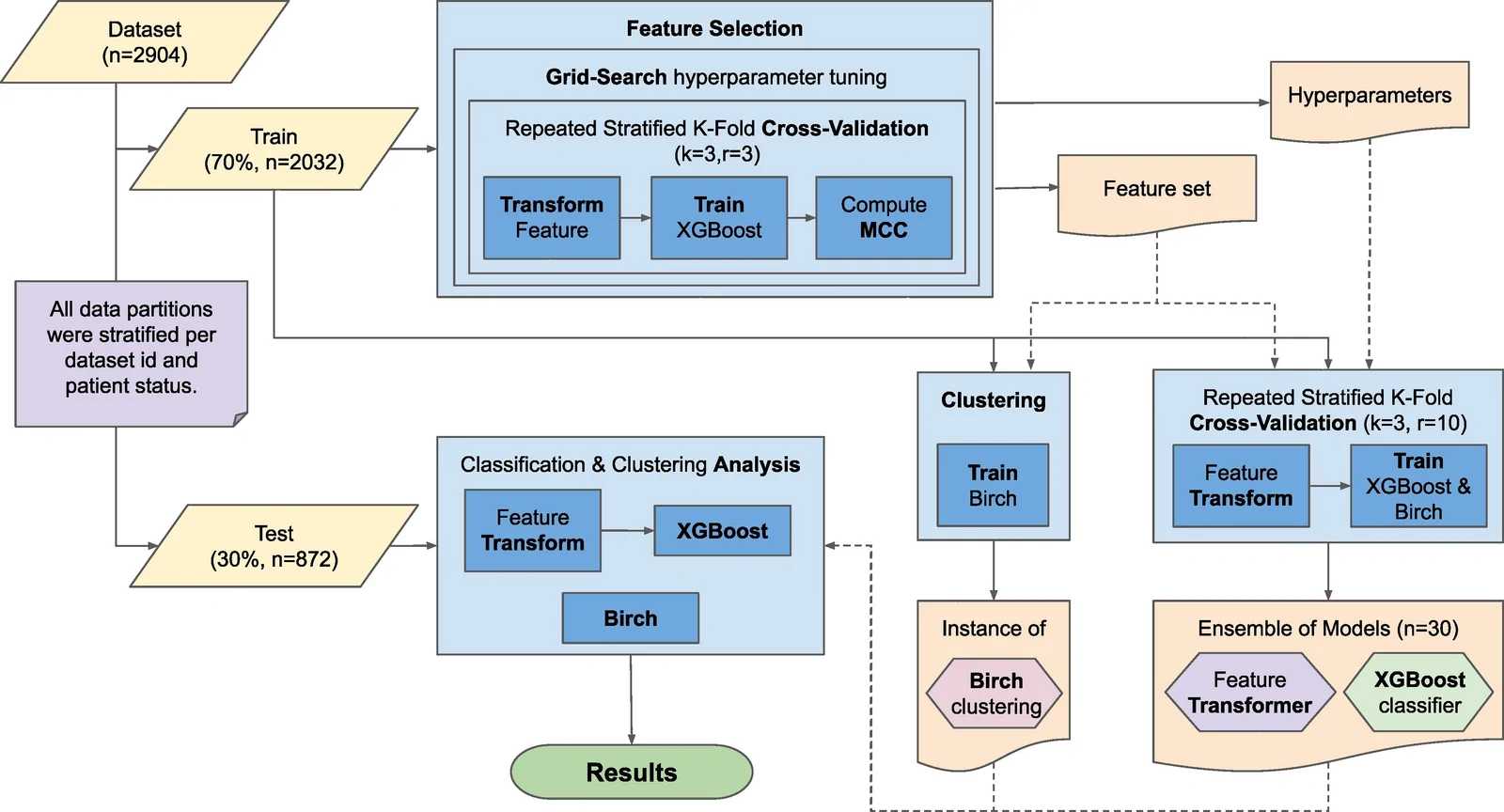
Computational methodology pipeline.

### Sample partitioning and training methodology

We stratified each dataset using the target variable, 1-year status MM or AT, into training (70%) and testing (30%). In the training stage, we performed feature selection and hyperparameter tuning of the XGBoost model using a cross-validation strategy. We use a variant of forward feature selection, in which the best feature set is found at each forward iteration and used as a base for the next iterations, continuing iterations until the last feature is tested. Note that this variation does not increase the computational complexity of the forward feature selection method and is useful for avoiding local optima. Regarding the grid search for XGBoost hyperparameters tuning, the search space was defined by four parameters (160 combinations), while the rest was empirically predefined. The complete list of hyperparameters and the grid-search ranges are detailed in Appendix 5. We applied a feature-wise power transformation to improve the distribution pattern of the data. This step helps reduce the impact of extreme values or outliers, ensuring the model can make more accurate predictions. By improving the normality of the data, the model can better understand the relationships between the variables, leading to more reliable results in data analysis. A zero-mean, unit-variance normalization is applied to the transformed data; see Appendix 5 for details on the computational implementation. The proposed methodology selects the feature set that maximizes Matthews correlation coefficient (MCC) found by the grid search through a repeated stratified k-fold cross-validation (folds k=3, repetitions r=3). The optimal feature set and the corresponding XGBoost hyperparameters are used for a final training using a repeated stratified k-fold cross-validation (k=3, r=10) over the training subsample, yielding 30 XGBoost models. These models are ensembled using an averaged prediction score to perform predictions on the test set.

All computations were performed on a standard personal computer, an 11th-generation Intel^®^ Core^™^ i7-1165G7 @2.80 GHz ×8 with 48 GB RAM. The ensembled model’s throughput is 0.0001 s per patient. The interested reader will find the developed model embedded within the ML4HF^™^ app, which is pending official release in the Apple APP Store^®^, see also www.ml4hf.app.

## Results

The feature selection and hyperparameter tuning stage used 2032 patients, with a prevalence of 17.57% of AT/D. The forward feature selection algorithm took as input the 14 haemodynamic features listed in *Table 1* and selected six, namely LVMPS, SPAP, DAP, LVCPO, RVMPS, and RVCPO (features reported in the order obtained from the forward feature selection algorithm; see Section ‘Sample partitioning and training methodology’). Regarding the XGBoost configuration, which resulted from the grid search of hyperparameters, the final model configuration is set to contain 16 estimators and 6 levels of depth. For a complete description, see Appendix 5. We report the predictive performance of the ensemble of 30 models produced by the repeated stratified k-fold cross-validation. In this way, it is possible to assess the variability in the predictions for the population of models. In the testing stage, 872 patients with a prevalence of 17.55% were used. Predictive statistics are presented in *Table 2*. *Figure 2* presents the receiver-operator (top left panel) and the precision-recall (top right panel) curves corresponding to the ensemble model (see Section ‘Machine learning model’). In the context of our analysis, SHAP values allow us to quantify the impact of each haemodynamic variable (such as myocardial performance score or diastolic arterial pressure) on the predictions made by the ensemble of XGBoost models. The higher the SHAP value for a feature, the more influence that feature has on the model’s decision. This is particularly useful for understanding which variables are most important for consistently predicting advanced outcomes like LVAD, heart transplant, or death in heart failure patients. By visualizing SHAP values in *Figure 2* (bottom panel), we can rank the features by their importance, giving us a clear picture of which haemodynamic factors drive the model’s predictions. This technique not only helps explain the model’s behaviour but also provides valuable insights for clinicians by highlighting the most relevant factors influencing patient outcomes and potential targets for therapy.

**Table 2:** Predictive capability statistics of the ensemble model over the 872 test patients, and also segregated per dataset

All (n=872, prevalence 17.55%)						
global	AUC	MCC	ACC	F1 avg		
metrics	0.81	0.35	0.74	0.65		
Class	SPE	SEN	PPV	NPV	F1 (MM)	F1 (AT/D)
metrics	0.76	0.67	0.37	0.92	0.83	0.48
Confusion	TN	FP	FN	TP		
matrix	543	176	50	103		
UCMC-SHOCK (n=58, prevalence 68.97%)						
Global	AUC	MCC	ACC	F1 avg		
metrics	0.83	0.33	0.74	0.64		
Class	SPE	SEN	PPV	NPV	F1 (MM)	F1 (AT/D)
metrics	0.33	0.93	0.76	0.67	0.44	0.83
Confusion	TN	FP	FN	TP		
matrix	6	12	3	37		
PREDICT-HF (n=178, prevalence 30.34%)						
Global	AUC	MCC	ACC	F1 avg		
metrics	0.68	0.15	0.6	0.57		
Class	SPE	SEN	PPV	NPV	F1 (MM)	F1 (AT/D)
metrics	0.64	0.52	0.38	0.75	0.69	0.44
Confusion	TN	FP	FN	TP		
matrix	79	45	26	28		
VUMC (n=636, prevalence 9.28%)						
Global	AUC	MCC	ACC	F1 avg		
metrics	0.8	0.29	0.78	0.61		
Class	SPE	SEN	PPV	NPV	F1 (MM)	F1 (AT/D)
metrics	0.79	0.64	0.24	0.96	0.87	0.35
Confusion	TN	FP	FN	TP		
matrix	458	119	21	38		

AUC, area under the curve; ACC, accuracy; F1, F1-score; FN, false negative; FP, false positive; MCC, Matthews correlation coefficient; NPV, negative predictive value; PPV, positive predictive value; SEN, sensitivity; SPE, specificity; TN, true negative; TP, true positive.

**Figure 2:**
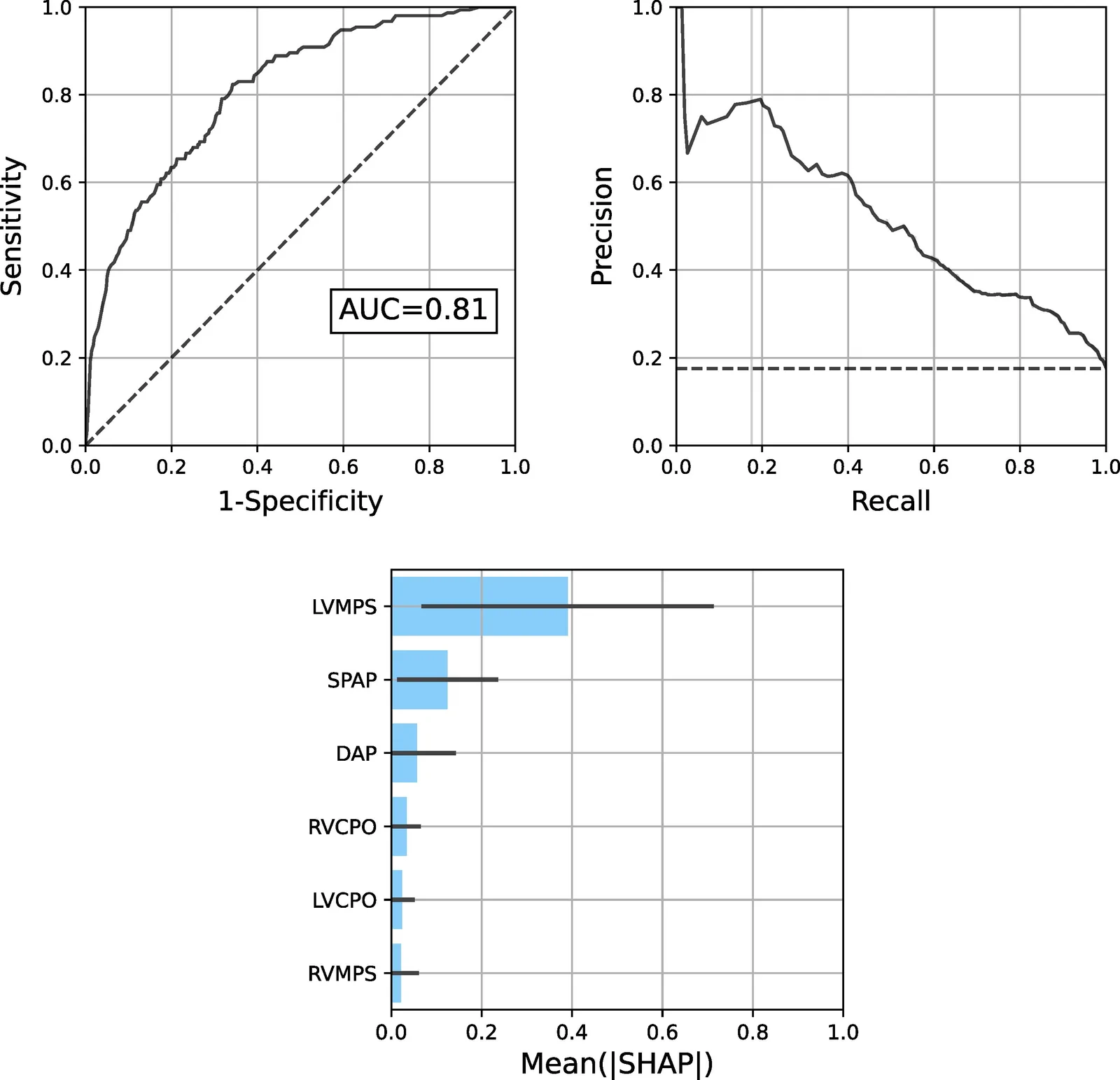
Receiver-operator curve (top left panel), precision-recall curve (top right panel), and mean SHapley Additive exPlanations (SHAP) values per feature (bottom panel). Metrics were computed over the test patients (n=872).

*Figure 3* presents boxplots describing the distributions of the haemodynamic variables and the energetic variables according to the ground truth class and to the class as predicted by the XGBoost model. Boxplots provide a complete characterization of the numerical data, featuring the median value (central line), the interquartile range Q3–Q1 (box limits), and the non-outlier data points (whiskers). Eventually, outliers are shown as individual points beyond the whisker limits. Skewness of the data distribution is also easily visualized through boxplots. It can be seen that no single haemodynamic and energetic biomarker is capable of discriminating the two hard endpoints (MM and AT/D). The results obtained using unsupervised clustering analysis through the BIRCH model are described in Appendix 4.

**Figure 3:**
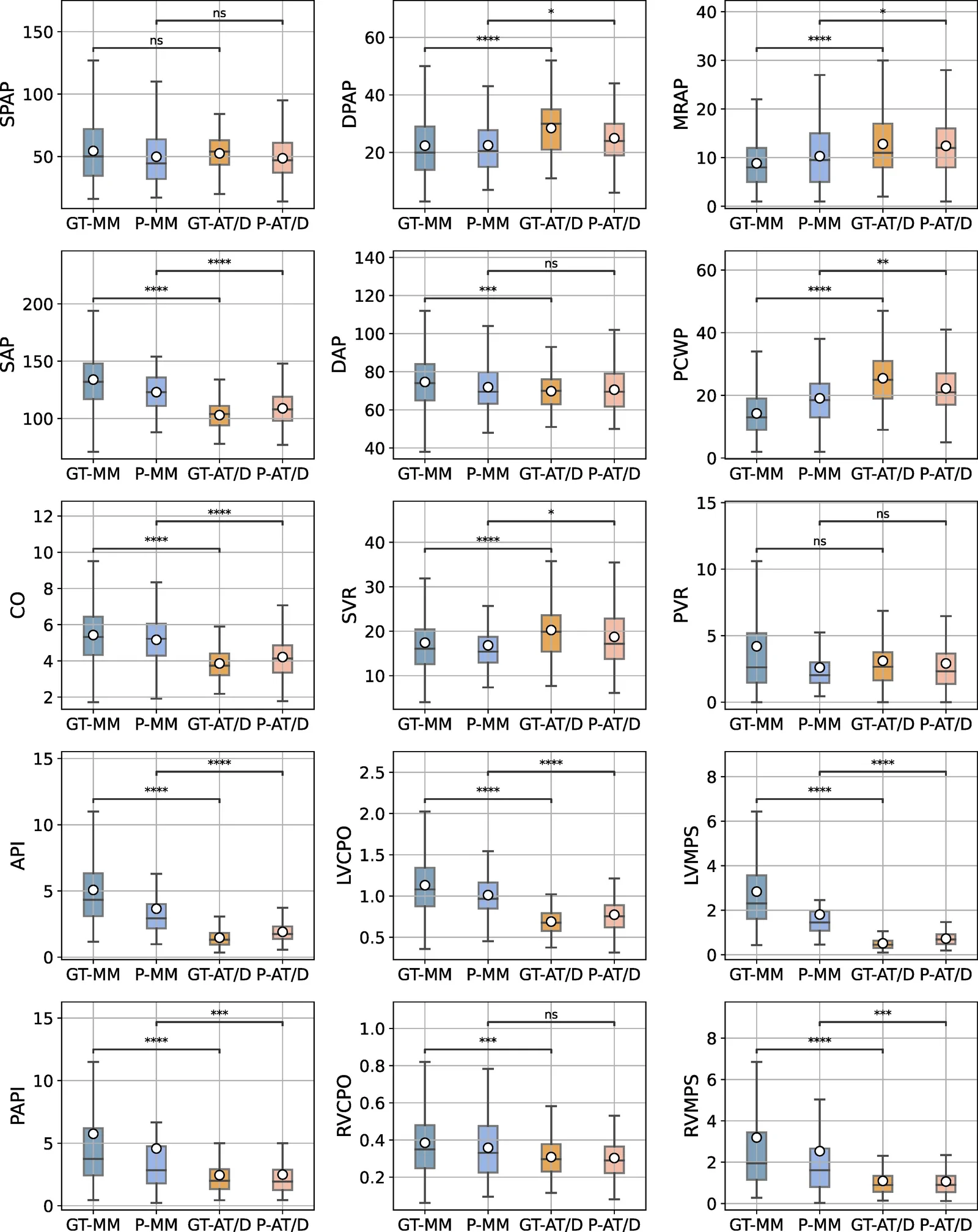
Boxplots of features segregated by the ground truth (GT) and by the predicted (P) medical management (MM) and advanced therapy/death (AT/D) classes on the test set. The white circle indicates the group means, and the markers indicate significant differences in mean values according to a non-parametric two-tailed Mann–Whitney U-test (p<0.05). Input variables for the XGBoost model are left ventricle myocardial performance score (LVMPS), systolic arterial pressure (SPAP), diastolic arterial pressure (DAP), left ventricle cardiac power output score (LVCPO), right ventricle myocardial performance score (RVMPS), and right ventricle cardiac power output score (RVCPO).

Finally, we report a result about the subgroup of patients for which the model predicted AT/D, but did not actually receive AT and died within 1 year. We call this group the group of ‘overlooked’ patients. According to our ML model, the number of patient whom the model predicted AT/D, and did not receive HT neither LVAD, and died within 1 year is 15 in the test set. This represents 1.72% of the size of the test set. The dataset breakdown is the following: 12 patients from the PREDICT-HF database, and 3 patients from the UCMC-SHOCK database.

## Discussion

### Impact of augmented care

Heart failure is a highly heterogeneous condition, with different signals of disease trajectory depending on the chronicity and mechanism of cardiac injury. Although numerous clinical risk prediction models have been developed over the past two decades, they have been ineffective at meaningfully delineating risk. Multivariate clinical risk scores—such as MAGGIC and SHFM—incorporate laboratory, demographic, and echocardiographic data, but were developed in previous eras of medicine prior to recent advances in device and medical therapies. In real-world systolic heart failure populations, these models have shown only modest predictive power, with AUC values for mortality ranging from 0.71 to 0.74.^9^ Notably, these scores do not incorporate haemodynamic data. Predictive models that rely largely or exclusively on haemodynamic data are limited; for example, cardiac power output, previously considered a strong haemodynamic predictor of mortality in acute MI shock, achieved an AUC of just 0.69 and a negative predictive value of 71%.^10^

We have shown that either individual biomarkers (see *Figure 3*) or standard/advanced haemodynamic variables introduced into logistic regression models (see Appendix 3) are unable to discriminate the risk of need for advanced therapy or death. Therefore, in this work we presented an integrative ML tool that combined advanced haemodynamic and energetic data collected in the cath lab to predict the need for heart transplant, LVAD implantation, or death with an AUC of 0.81 and a negative predictive value of 92%. Our XGBoost-based model performs promisingly for predicting hard end-point outcomes in end-stage heart failure patients, starting from haemodynamic and energetic inputs. Previous studies utilizing conventional logistic regression models—based on administrative claims and electronic health records—report AUC–ROC values ranging from 0.74 to 0.80, with little gains in predictive accuracy even when more clinical data are incorporated.^11,12^

These models typically use composite endpoints that are largely driven by heart failure hospitalizations or functional improvement. In contrast, our model achieved robust prediction of hard endpoints, such as the need for advanced therapy or death. While other machine learning approaches have leveraged longitudinal electronic health record data to predict new-onset heart failure or readmissions,^13,14,15^ the proposed approach is novel in that it integrates haemodynamic data through cardiac energetics which enhances the predictive accuracy. These findings underscore the added value of combining machine learning with detailed physiological data to improve outcome predictions in heart failure management to aid clinical decision-making. Furthermore, the algorithm presented here is able to interpret haemodynamic and energetic profiles of patients irrespective of age and gender, adding to its application to patients across all spectrums of heart failure.

### Myocardial energetics and hemodynamic outcomes

Myocardial energetics play a critical role in the overall function and efficiency of the heart, particularly in heart failure. Cardiac energetics refers to the balance between the energy supply available to the myocardium and its mechanical workload, essentially the heart’s ability to convert biochemical energy into mechanical output.^16,17^ In a failing heart, this balance is often disrupted, leading to diminished myocardial efficiency and worsening clinical outcomes. The link between myocardial energetics and hemodynamics is well established, as poor energy utilization impairs contractile function, leading to increased cardiac filling pressures, reduced cardiac output, elevated pulmonary artery pressures, and potential end-organ damage. The net result is a loss of mechanical efficiency, defined as the ratio of ventricular stroke work to overall ventricular work. Simultaneously, there is a progressive uncoupling of the ventriculo-arterial unit, leading to further reductions in efficiency.

### Importance of haemodynamic variables

Our analysis demonstrated that haemodynamic variables are a strong factor in the importance of our predictive power. This is consistent with previous studies that emphasize the importance of detailed haemodynamic monitoring in end-stage heart failure management. Considering the full set of myocardial energetics features, LVMPS, LVCPO, RVMPS, and RVCPO metrics were selected together with the haemodynamic variables, such as SPAP and DAP, bolstering the model performance. Adding information that encodes a level of intrinsic myocardial reserve has not been seen in the practice of managing heart failure patients until now. This unmasking of potential energy within the myocardium correlates with the clinical deterioration of patients as they progress to further loss of ventricular-aortic coupling. Using SHAP values, we were able to interpret the contributions of these variables, offering transparency in the model’s predictions, which is critical for clinical integration. Explainable AI approaches like SHAP improve the trust and utility of machine learning models in clinical settings by making the decision-making process clear to clinicians. Specifically, the myocardial performance score (MPS) was the largest driver of the model’s accuracy. The MPS represents the power efficiency of the left ventricle and strongly correlates with mechanical efficiency, coupling, and clinical outcomes.^1^ The use of standard haemodynamic variables routinely obtained during right heart catheterization makes this algorithm highly accessible to clinicians at centres equipped to perform these procedures. By relying on universally available clinical data, this model can be widely implemented across various healthcare settings, ensuring that future iterations of the algorithm can provide valuable insights for heart failure management on a global scale.

Moreover, regarding the model selection phase, it is also worth mentioning that although we reported results for the XGBoost model in the previous section, the logistic regression model delivered comparable results in the training phase. The systematic model selection strategy based on the MCC pointed to XGBoost as the definitive choice, and so we have not assessed the performance of the logistic regression model in the test case to conclusions driven by data leak. However, based on the training performance, it is possible to say that logistic regression using standard haemodynamic variables is also able to deliver satisfactory performance. This is because the advanced haemodynamic variables emerge from nonlinear combinations of the standard ones. In spite of this, we believe the MPS’s relevance in the variable importance revealed by the SHAP analysis indicates the significance of advanced haemodynamic variables in characterizing patient risk. In other words, the relevance of MPS already proven in clinical practice^1^ is also highlighted by the SHAP analysis of the XGBoost model.

### Clustering and patient subgroup analysis

The Birch clustering algorithm was employed to identify subgroups within the patient population. However, variability in the prevalence of advanced therapies across different folds suggested that these clusters may not be robust enough for direct clinical application. This variability aligns with challenges noted in unsupervised learning techniques when applied to complex clinical datasets.^18^ Four clusters were identified with clusters A and C having both the highest prevalence as well as the best overall accuracy. Both clusters A and C represent patients with biventricular elevations in filling pressures, whereas cluster A is associated with a reduction in biventricular myocardial performance (cold and wet) and cluster C has preserved biventricular performance with a notably robust right ventricle. Cluster A had the highest event rates, and cluster C had the lowest event rates. These observations support the concept that congestion alone can have mixed prognostic value and added energetic information in the form of the myocardial performance score can provide additional insight into a patient’s trajectory. Clusters B and D represent patients with lower intracardiac filling pressures, relative right ventricle under-performance (cluster B) and lower systemic vascular resistance (cluster D), and thus, part of the lower prognostic yield of these cohorts might be related to mixed physiological disease states in these clusters. Additionally, given the balance of deranged haemodynamics and poor myocardial efficiency, some patients may fall within a continuum based on time-dependent analysis of hemodynamics, further emphasizing the need for dynamic assessment based on applied therapeutics for managing these complex patients. XGBoost was chosen as the primary focus of our predictive model due to its proven effectiveness in handling complex, high-dimensional datasets like those seen in heart failure management. This algorithm excels in processing structured data by building multiple decision trees and using a gradient-boosting framework to optimize predictive accuracy. During our model selection process, XGBoost slightly outpaced other machine learning algorithms (support vector machines, multi-layer perceptron, and Random Forest) in cross-validation tests (results not reported here), showing superior results in metrics such as accuracy, AUC–ROC, and negative predictive value. Still, the estimation of confidence intervals and the performance in real-world external validation studies is to be checked. Its ability to handle missing data, scalability, and robustness in avoiding overfitting made it the ideal choice for our development process,^3,19^ providing more consistent and reliable predictions than unsupervised techniques like clustering.

### Clinical applications

Currently, nearly half of patients who are eligible for heart replacement therapy are referred outside of the ‘Golden Window’ and as such, many are either deemed ineligible for therapies and/or present in multi-organ failure, complicating subsequent interventions.^20^ The haemodynamic assessment remains ubiquitous in clinical cardiology care, yet meaningful data interpretation remains elusive for many clinicians. United Network of Organ Sharing (UNOS) guidelines, and clinicians alike, still place excessive prognostic weight on individual haemodynamic parameters such as cardiac output despite mounting evidence that in isolation, cardiac output lacks the prognostic signal in chronic heart failure, and heart failure-related shock.^4,21,22^ This vulnerable group has the most unmet need and is a source of the delayed referral patterns discussed above. While end-organ delivery might be preserved when cardiac output is sufficient, this myopic view ignores the conditions that allowed the heart to generate the degree of output. A heart operating at the flat part of the Starling curve to generate a given degree of cardiac output is inherently sicker than a heart that is able to generate the same flow while operating on a steeper part of their Starling curve. This information is reflected by API and MPS but not CO, CI, or CPO alone.^23^ An interesting observation can be made when examining the 15 patients (1.7% of the cohort) where the model predicted AT/D but where patients did not receive an LVAD or HT and ultimately died. The majority of these patients (N=12) came from the PREDICT-HF cohort which was a less sick cohort than the UCMC-SHOCK (N=3) cohort. This highlights the importance of enhanced analytic and prognostic needs to identify residual risk in earlier stages of heart failure to allow for more timely recognition of early clinical decline. Risk was particularly underappreciated in the PREDICT-HF cohort which had more power reserve as demonstrated by higher systemic output metrics including CO (4.1 vs. 3.5 L/min) and CPO (0.73 vs. 0.60) which might have influenced decision making in real time. By definition of the selection criterion, our model identified risk appropriately for these 15 patients, for which the traditional assessment failed. These patients were generating output and power but at an energetic cost which was not-sustainable. By objectively defining risk, our algorithm can be directly implemented into multidisciplinary transplant and LVAD meetings to help objectively define Stage D heart failure and the need for heart replacement therapy.

A ML algorithm that is entirely rooted in the haemodynamic assessment offers complementary and additive prognostic information to other integrated risk tools: (1) by integrating advanced haemodynamic and energetic information, our model inputs reflect the drivers of pathophysiology and not bystanders of pathology and thus should be more reproducible when applied to diverse datasets; (2) utilization of haemodynamic data, exclusively, without the need for concomitant demographic, laboratory, or imaging data allows for a closed-loop integration and application of our algorithm into cath lab software and bedside haemodynamic monitoring devices. Furthermore, as wearable and implantable haemodynamic monitoring systems continue to evolve, the haemodynamic-focused inputs of our model will allow for adaptation to the ambulatory setting.

### Limitations and future directions

While our model achieved significant predictive accuracy, further validation across larger datasets to ensure the generalizability of our findings is key. The databases employed featured different HF conditions, from mild to moderate (VUMC and PREDICT-HF), and to severe (UCMC-SHOCK). Assembling a representative database containing the entire HF spectrum is a challenge, and would be a better approach to train a model that learns from the full patient spectrum of the HF condition. Hence, true external validation is a necessary step to provide more robust evidence of the capabilities of the proposed ML model in real-world scenarios.

Given the retrospective nature of this analysis, it is possible that clinician bias or misinterpretation of the haemodynamic data in real-time influenced the decision to proceed or not proceed with LVAD or transplant. The combined endpoint of LVAD, transplant or death partially mitigates internal bias as mortality is a competing risk for patients who were not offered LVAD or transplant due to misinterpretation of the data. Patients whose risk was underappreciated in real-time and not offered LVAD or transplant would be expected to still be captured in the combined endpoint. Conversely, patients whose risk was overappreciated will lead to a higher event rate. Events rates were higher using our predictive algorithm when compared to individual haemodynamic inputs such as CI and PCWP (variables that were being considered at the time of transplant or LVAD eligibility) speaking against wide-spread systemic over-estimation of true risk.

Currently, RHC measurements used as input variables are static (one time point) rather than dynamic, which would be ideal for HF patients. However, more advanced machine learning techniques and direct integration of automated waveform analysis of haemodynamic tracings will be explored in future work. Finally, the definition of the 1-year follow-up is a matter of discussion. The available data enabled us to perform this study as a preliminary investigation to determine the feasibility of developing ML models to predict future outcomes from baseline data. Further statistical analyses involving prospective data and confidence interval estimation must follow before clinical translation.

## Conclusion

Combining standard haemodynamic variables with advanced patient energetics as input variables to a machine learning model provides a new pathway for more precise clinical decision-making in managing heart failure, particularly in identifying high-risk patients who may benefit from consideration of advanced therapies. The proposed model featured a promisingly high negative predictive value. Translating it into clinical routine must be preceded by external validation and prospectively collected databases.

## Figures and Tables

**Figure C1:**
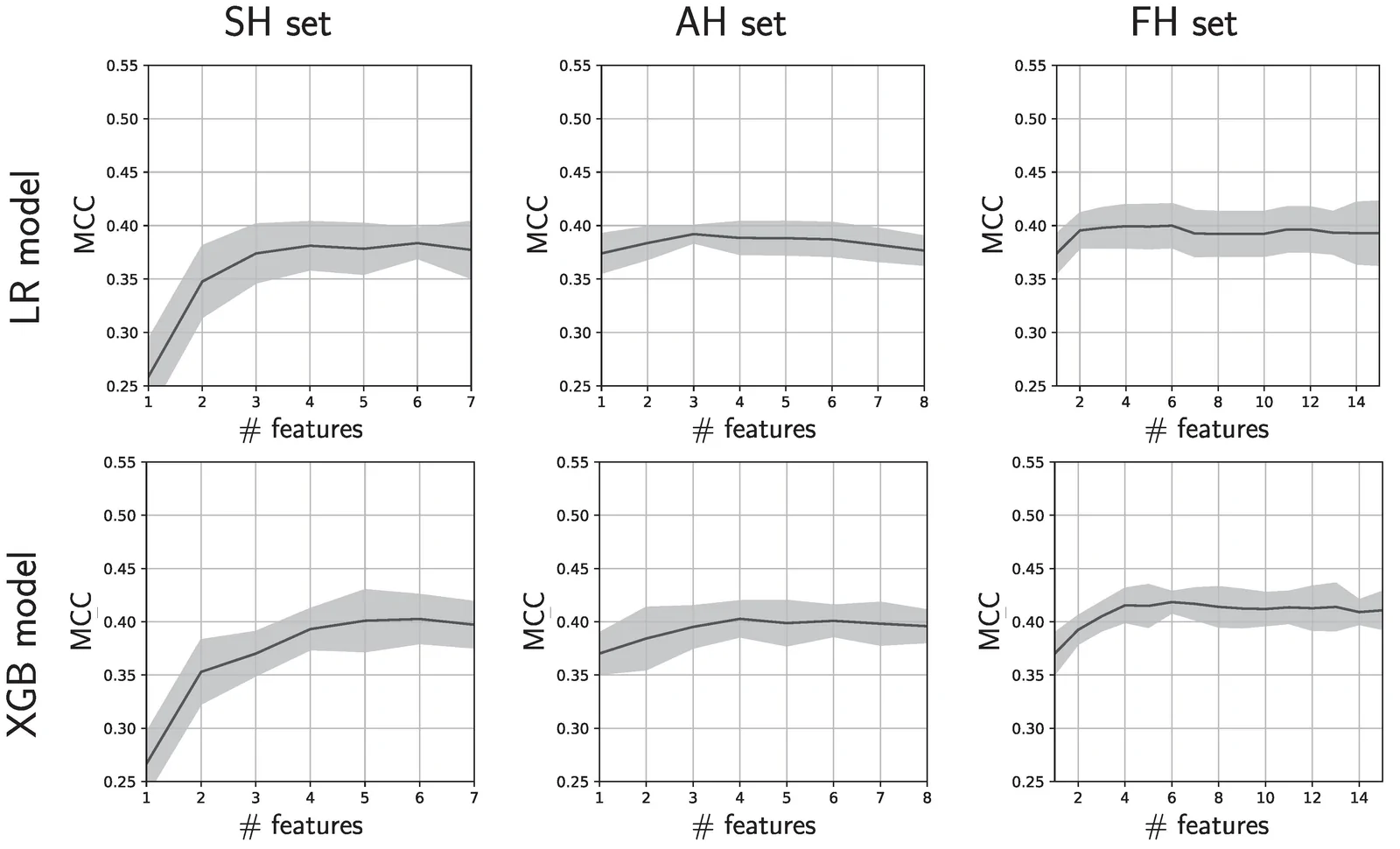
Matthews correlation coefficient (MCC, solid line represents the mean value, and shaded area is the standard deviation) for the best model configuration at each iteration of the forward feature selection procedure. The first row displays the experiments with the logistic regression model, and the second row displays the results for the XGBoost model. From left to right, columns correspond to the SH, AH, and FH =SH+AH feature sets.

**Figure D1:**
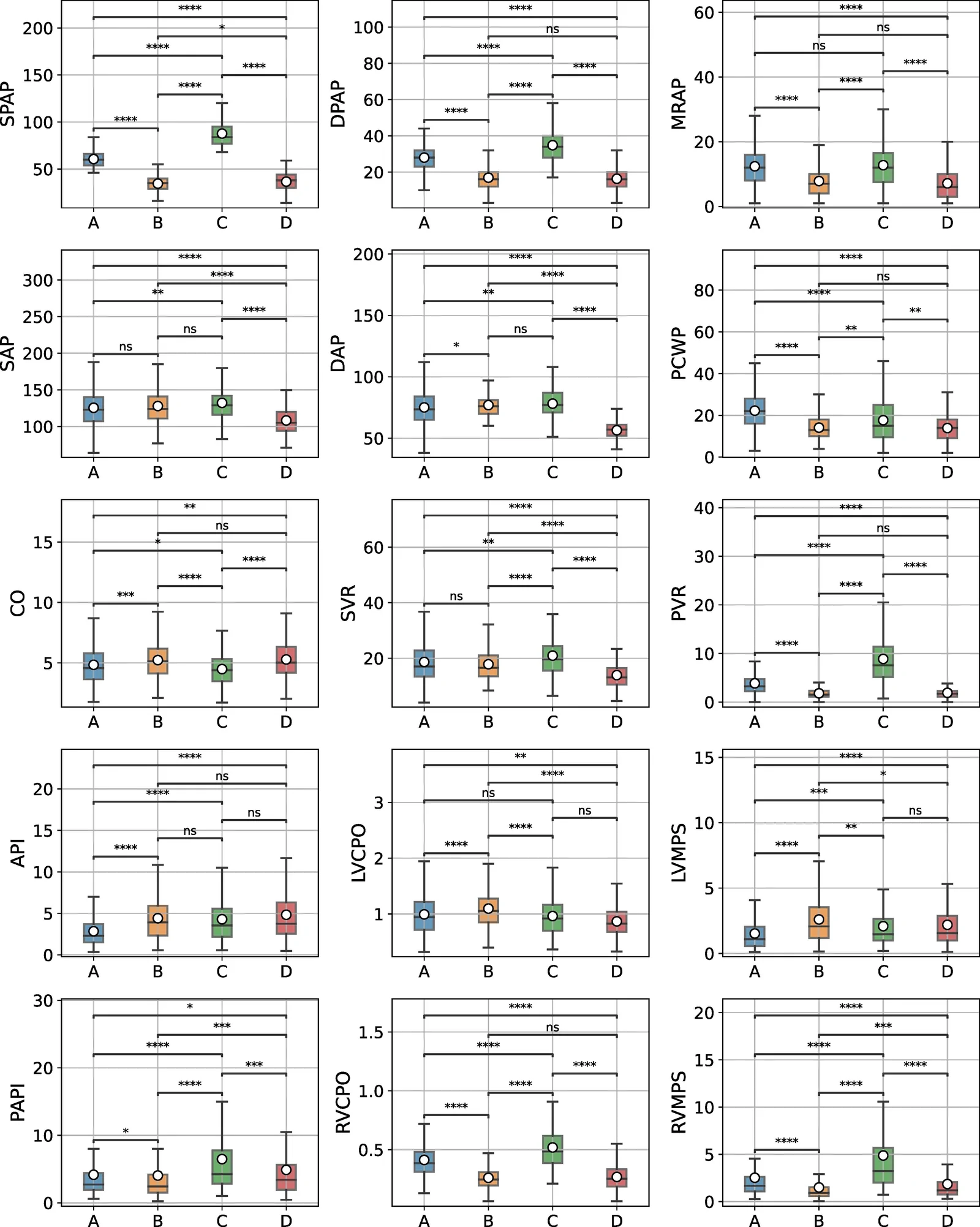
Boxplots of all variables of the test set grouped by cluster (A, B, C, D). Input variables for the BIRCH model are left ventricle myocardial performance score (LVMPS), systolic arterial pressure (SPAP), diastolic arterial pressure (DAP), left ventricle cardiac power output score (LVCPO), right ventricle myocardial performance score (RVMPS), and right ventricle cardiac power output score (RVCPO).

## Data Availability

The data underlying this article cannot be shared publicly due to origin of the data being derived from multiple institutions with data sharing restrictions. Specific requests will be managed by the corresponding author.

## Appendix 1 Abbreviations

- API: aortic pulsatility index
- AT: advanced treatment
- CO: cardiac output
- DAP: diastolic arterial pressure
- DPAP: diastolic pulmonary artery pressure
- HT: heart transplant
- LVAD: left ventricle assist device
- LVCPO: left ventricle cardiac power output score
- LVMPS: left ventricle myocardial performance score
- MAP: mean arterial pressure
- MM: medical management
- MPAP: mean pulmonary artery pressure
- MRAP: mean right atrial pressure
- PAPI: pulmonary artery pulsatility index
- PCWP: pulmonary capillary wedge pressure
- PVR: pulmonary vascular resistance
- RHC: right heart catheterization
- RVCPO: right ventricle cardiac power output score
- RVMPS: right ventricle myocardial performance score
- SAP: systolic arterial pressure
- SPAP: systolic pulmonary artery pressure
- SVR: systemic vascular resistance

## Appendix 2 Formulae

$MAP=\frac{2\;\text{DAP}+\text{SAP}}{3},\;\text{MPAP}=\frac{2\;\text{DPAP}+\text{SPAP}}{3},$ $API=\frac{\text{SAP}-\text{DAP}}{\text{PCWP}},\text{PAPI}=\frac{\text{SPAP}-\text{DPAP}}{\text{MRAP}},$  $LVCPO=\frac{\text{MAP}\cdot \text{CO}}{451},\text{RVCPO}=\frac{\text{MPAP}\cdot \text{CO}}{451},$  $LVMPS=\frac{1}{2}\;\text{API}\cdot \text{LVCPO},\text{RVMPS}=\frac{3}{2}\;\text{PAPI}\cdot \text{RVCPO},$  $\text{SVR}=\frac{\text{MAP}-\text{MRAP}}{\text{CO}},\text{PVR}=\frac{\text{MPAP}-\text{PCWP}}{\text{CO}},$

## Appendix 3 Model selection

In this section, we compare the performance of the proposed XGBoost model, with that delivered by logistic regression models as a strategy to justify model selection by looking into the incremental benefit derived from both the more advanced modelling technique and the combined feature set. We also evaluated other models, including Random Forest, support vector machine, and multi-layer perceptron, but these results are not reported here for the sake of conciseness.

From the features listed in *Table 1*, we considered the following sets:

- Standard haemodynamic (SH) set:
  SH={PCWP,DAP,SAP,CO,MRAP,DPAP,SPAP}
- Advanced haemodynamic (AH) set (see Appendix 2):
  $\begin{array}{r} \text{AH}=\{\text{API},\text{LVCPO},\text{LVMPS},\text{PAPI},\\ \text{RVCPO},\text{RVMPS},\text{SVR},\text{PVR}\} \end{array}$
- Full haemodynamic (FH) set:
  FH=SH+AH

Then, we execute our training pipeline (see *Figure 1*), consisting of feature selection, grid search for hyperparameter tuning, and repeated stratified cross-validation (see Section ‘Sample partitioning and training methodology’ for details). We utilized the identical training dataset and ensured the same partitions during cross-validation to ensure a fair comparison. During this process, we evaluated the LR model and the XGB model fed with the three different sets described above.

Note that for XGBoost, the base configuration and the grid search space of hyperparameters were the same as those reported in the original manuscript. For logistic regression, the base configuration and the grid search space are as follows.

sklearn.linear_model.LogisticRegression(

penalty=“elasticnet”,

C=1.0,

l1_ratio=0.0,

dual=False,

tol=0.0001,

fit_intercept=True,

intercept_scaling=1,

class_weight=None,

random_state=0,

solver=“saga”,

max_iter=200,

verbose=0,

warm_start=False,

n_jobs=None)

parameters = {

“C”: [0.01, 0.5, 1.0, 1.5, 2, 8],

“l1_ratio”:  [0.0, 0.2, 0.4, 0.6, 0.8, 1.0],

“class_weight”: [None, “balanced”] }

*Figure C1* presents the mean and STD (over the nine models of the three-fold cross-validation with three repetitions for the model selection stage) for the model configured through grid-search parameter that resulted in the highest MCC at each incremental addition of feature during the forward feature selection. Coefficients, hyperparameters, and feature subsets are presented in *Table C1*.

The results of this analysis illustrate two relevant conclusions:

1. The SH set resulted in lower predictive capabilities for both logistic regression and XGBoost models. Also, although the AH set improves the predictive capability of both models, the best results are obtained when the FH set of features is used.
2. The XGBoost consistently resulted in better predictive capabilities, regardless of the feature set.

This rigorous comparison confirms that both the use of the XGBoost architecture and our chosen feature selection methodology from the FH set meaningfully contribute to improved predictive performance over simpler logistic regression models using the SH set.

We have compared the models only using the training partition. After concluding that the selection of the model-features combination must be XGBoost-FH, we proceeded to evaluate the predictive capabilities of this selected model in the main body of the manuscript. Note that we did not compare the performance of the models on the test set, as it would produce data leak in the model selection procedure.

**Table C1:** Hyperparameter values found during the grid-search and the resulting feature list (ordered as they were found in the forward feature selection process) for each scenario and model

Model/Set	MCC	AUC	Ordered feature list	Hyperparameters
LR / SH	0.38 (0.01)	0.81 (0.01)	SAP, PCWP, CO, MRAP, DPAP, DAP	C=1.0, class_weight=‘balanced’, l1_ratio=0.4
LR / AH	0.39 (0.01)	0.81 (0.01)	LVMPS, RVCPO, LVCPO	C=0.01,
				class_weight=‘balanced’,
				l1_ratio=0.2
LR / FH	0.40 (0.02)	0.82 (0.02)	LVMPS, SPAP, MRAP, CO, RVMPS, PVR	C=0.5,
				class_weight=‘balanced’,
				l1_ratio=1.0
XGB / SH	0.40 (0.02)	0.82 (0.01)	SAP, PCWP, CO, SPAP, DAP, DPAP	learning_rate=0.125,
				max_delta_step=1,
				max_depth=4,
				n_estimators=64,
				scale_pos_weight=0.25
XGB / AH	0.40 (0.02)	0.82 (0.01)	LVMPS, PVR, SVR, RVMPS	learning_rate=0.125,
				max_delta_step=0,
				max_depth=2,
				n_estimators=32,
				scale_pos_weight=0.25
XGB / FH	0.42 (0.01)	0.82 (0.01)	LVMPS, SPAP, DAP, LVCPO, RVMPS, RVCPO	learning_rate=0.125,
				max_delta_step=1,
				max_depth=4,
				n_estimators=32,
				scale_pos_weight=0.25

Also, the best MCC (mean and STD) for each case is reported; see Appendix 1 for abbreviations.

## Appendix 4 Unsupervised clusterization analysis

BIRCH is a scalable clustering algorithm designed for large datasets. Through incremental steps, it builds a hierarchical data structure, a clustering feature tree, that summarizes groups of data points using compact statistical representations. This allows the algorithm to process data efficiently, often in a single pass. Final clustering is then performed on these summaries rather than the raw data, significantly reducing computational cost while preserving the underlying data structure.

The ensemble of XGBoost models and the BIRCH model is used to predict and cluster the test dataset for the main analysis. BIRCH clustering is utilized in this methodology to identify meaningful subgroups within the patient population based on their haemodynamic and energetic profiles. It allows for discovering natural clusters in the data and grouping patients with similar characteristics, which may not be immediately obvious through other analytical methods. By identifying these clusters, we gain additional insights into how different groups of patients may have clinical progression of heart failure, and potentially respond to end-stage heart failure therapies.

The clustering analysis showed that clusters A and B contain approximately the same number of patients, which is twice as large as the number of patients in clusters C and D. Cluster A presents the largest prevalence of AT/D patients (23%), which represents an increase of roughly 50% in the prevalence of clusters C and D (10% and 14% each). *Table D1* reports the patient counts and distributions, segregated per class and cluster. See Appendix for details on the BIRCH algorithm configuration. These results suggest that a simple clustering analysis has less predictive value than the XGBoost model for identifying AT/D patients. Moreover, in *Table D2*, we present the predictive capability of the ensemble of XGBoost models segregated by cluster. The haemodynamic profile of cluster A aligns with classical biventricular heart failure, as there is a biventricular elevation in filling pressures and near symmetrical reduction in the left-sided and right-sided indices of efficiency (API and PAPI) and power-efficiency (MPS and RV-MPS). Cluster B and cluster D have lower filling pressures (RA and PCWP), with cluster B having reduced RV performance (reduced PA pulsatility, PAPI, RV-CPO, and RV-MPS) relative to LV performance, while cluster D driving phenotype is a low SVR state secondary to systemic vasoplegia. Cluster C, compared to cluster B, has elevated filling pressures and good RV performance. *Figure D1* presents boxplots of haemodynamic and energetic variables grouped by cluster.

**Table D1:** Patient count segregated per class and cluster according to the BIRCH model

	Cluster				
Class	A	B	C	D	Total
MM	228	234	132	125	719
AT/D	70	47	15	21	153
Total	298	281	147	146	872
Target distr.	A	B	C	D	Total
MM	0.32	0.33	0.18	0.17	1.00
AT/D	0.46	0.31	0.10	0.14	1.00
Total	0.34	0.32	0.17	0.17	1.00
Prevalence	A	B	C	D	Total
MM	0.77	0.83	0.90	0.86	0.82
AT/D	0.23	0.17	0.10	0.14	0.18
Total	1.00	1.00	1.00	1.00	1.00

See Appendix 1 for abbreviations.

**Table D2:** Predictive capability statistics of the ensemble model over the 872 test patients, segregated by each of the four BIRCH clusters

Cluster A (n=298, prevalence 23.49%)						
Global	AUC	MCC	ACC	F1 avg		
metrics	0.84	0.43	0.72	0.68		
Class	SPE	SEN	PPV	NPV	F1 (MM)	F1 (AT/D)
metrics	0.69	0.81	0.45	0.92	0.79	0.58
Confusion	TN	FP	FN	TP		
matrix	157	71	13	57		
Cluster B (n=281, prevalence 16.73%)						
Global	AUC	MCC	ACC	F1 avg		
metrics	0.73	0.25	0.71	0.61		
Class	SPE	SEN	PPV	NPV	F1 (MM)	F1 (AT/D)
metrics	0.74	0.57	0.31	0.9	0.81	0.4
Confusion	TN	FP	FN	TP		
matrix	173	61	20	27		
Cluster C (n=147, prevalence 10.20%)						
Global	AUC	MCC	ACC	F1 avg		
metrics	0.89	0.34	0.86	0.67		
Class	SPE	SEN	PPV	NPV	F1 (MM)	F1 (AT/D)
metrics	0.91	0.47	0.37	0.94	0.92	0.41
Confusion	TN	FP	FN	TP		
matrix	120	12	8	7		
Cluster D (n=146, prevalence 14.38%)						
Global	AUC	MCC	ACC	F1 avg		
metrics	0.78	0.24	0.72	0.59		
Class	SPE	SEN	PPV	NPV	F1 (MM)	F1 (AT/D)
metrics	0.74	0.57	0.27	0.91	0.82	0.37
Confusion	TN	FP	FN	TP		
matrix	93	32	9	12		

See Appendix 1 for abbreviations.

## Appendix 5 Computational implementation

The computational implementation was developed in Python v3.12, with the use of the following packages:

- numpy (https://github.com/numpy/numpy) and pandas (https://github.com/pandas-dev/pandas) for management of tabular data;
- scipy (https://github.com/scipy/scipy) for statistics;
- scikit-learn (https://github.com/scikit-learn/scikit-learn) for cross-validation, clustering, and classification performance metrics;
- xgboost (https://github.com/dmlc/xgboost) for the implementation of the XGBoost classifier;
- shap (https://github.com/slundberg/shap) for computing SHAP values.

We employ the Power Transformation feature-wise, with default parameters, to improve the normality of data distributions. A zero-mean, unit-variance normalization is applied to the transformed data.

ftrans = PowerTransformer(

method=‘yeo-johnson’,

standardize=True)

The XGBoost classifier was configured as follows:

xgboostreg = xgboost.XGBClassifier(

tree_method = “auto”,

scale_pos_weight=1.0,

n_estimators= 64,

max_depth= 6,

learning_rate= 0.125,

subsample= 0.98,

colsample_bytree= 0.9,

colsample_bylevel= 0.9,

colsample_bynode= 1.0,

objective= “binary:logistic”,

booster= “gbtree”,

use_label_encoder= false,

eval_metric= [“logloss”, “auc”],

random_state= 1,

verbosity 1,

reg_lambda= 1,

reg_alpha= 0,

max_delta_step= 0,

importance_type= “gain”)

Moreover, the Grid-Search algorithm explored the following XGBoost parameter value combinations:

parameters = {n_estimators}: [16, 32, 64, 128],

“scale_pos_weight”: [0.25, 0.5, 1.0, 1.25, 2.5],

“max_depth”: [2, 4, 6, 8],

“max_delta_step”: [0, 1] }

The configuration found by the grid-search procedure (with cross-validation) for the feature set that was selected was:

n_estimators = 32

scale_pos_weight = 0.25

max_depth = 4

max_delta_step = 1

The Birch algorithm was configured as follows:

birch = sklearn.cluster.Birch(

n_clusters=4,

threshold=0.5,

branching_factor=50,

compute_labels=True,

copy=True)
